# Supplementary material for: The impact of 22q11.2 copy-number variants on human traits in the general population
Source: Am J Hum Genet. 2023 Jan 26;110(2):300–13. doi: 10.1016/j.ajhg.2023.01.005 (PMC9943723; doi:10.1016/j.ajhg.2023.01.005)
Supplement: Document S1. Figures S1–S4 [file mmc1.pdf]

The American Journal of Human Genetics, Volume *110*

Supplemental information

The impact of 22q11.2 copy-number variants on human traits in the general population

Malú Zamariolli, Chiara Auwerx, Marie C. Sadler, Adriaan van der Graaf, Kaido Lepik, Tabea Schoeler, Mariana Moysés-Oliveira, Anelisa G. Dantas, Maria Isabel Melaragno, and Zoltán Kutalik

Supplemental Figures and legends

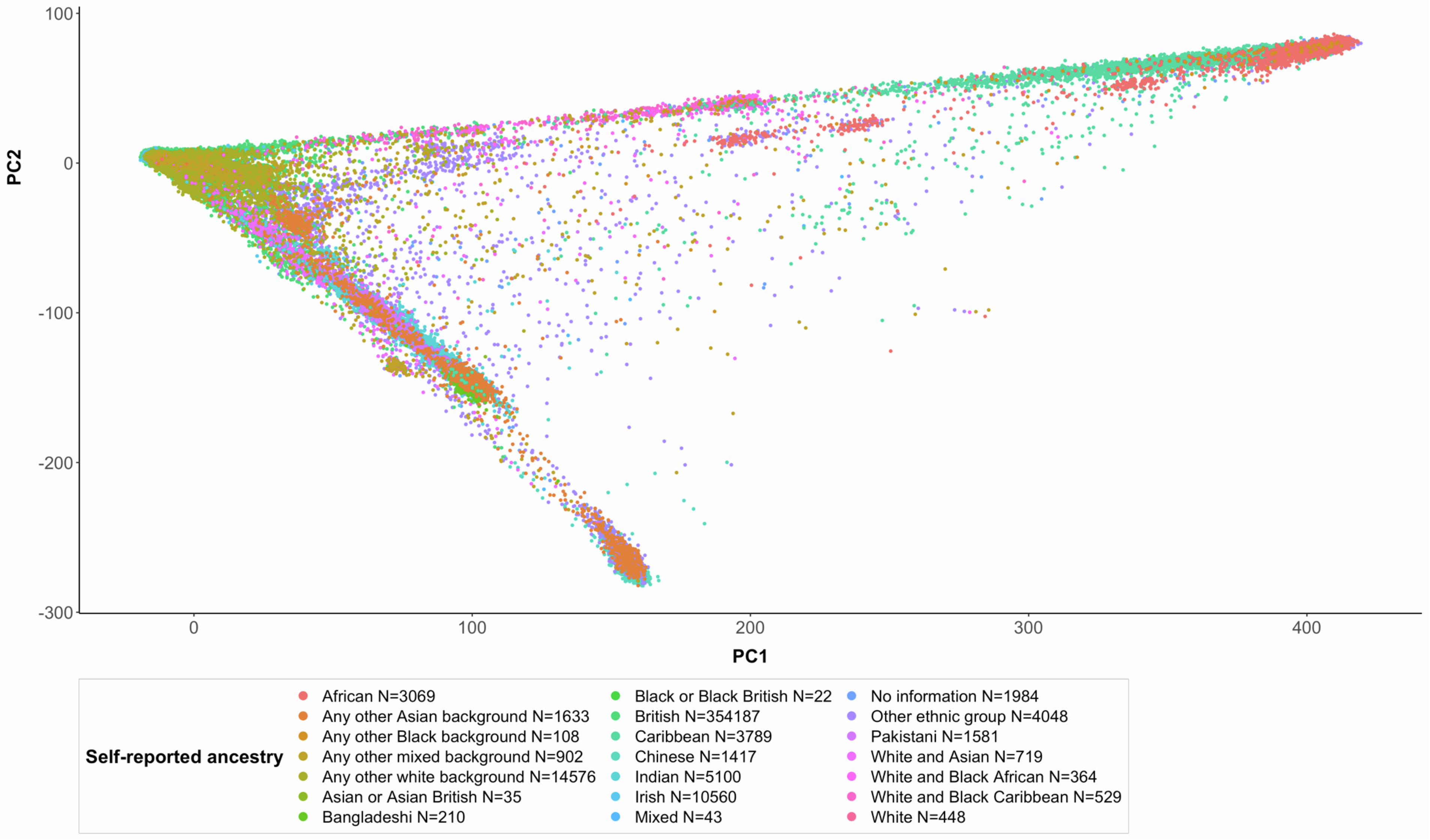
100
0
-100
-200
-300
PC2
0
100
200
300
400
PC1
Self-reported ancestry
African N=3069
Any other Asian background N=1633
Any other Black background N=108
Any other mixed background N=902
Any other white background N=14576
Asian or Asian British N=35
Bangladeshi N=210
Black or Black British N=22
British N=354187
Caribbean N=3789
Chinese N=1417
Indian N=5100
Irish N=10560
Mixed N=43
No information N=1984
Other ethnic group N=4048
Pakistani N=1581
White and Asian N=719
White and Black African N=364
White and Black Caribbean N=529
White N=448

Figure S1 | Scatterplot of the first two principal components (PC1 vs. PC2) of UKBB individuals. The values for the second principal component (PC2) are plotted against the values for the first principal component (PC1) for the UKBB individuals evaluated in the study. Points are colored based on self-reported ancestry. Total number of individuals (N) per ancestry is reported with the plot legend.

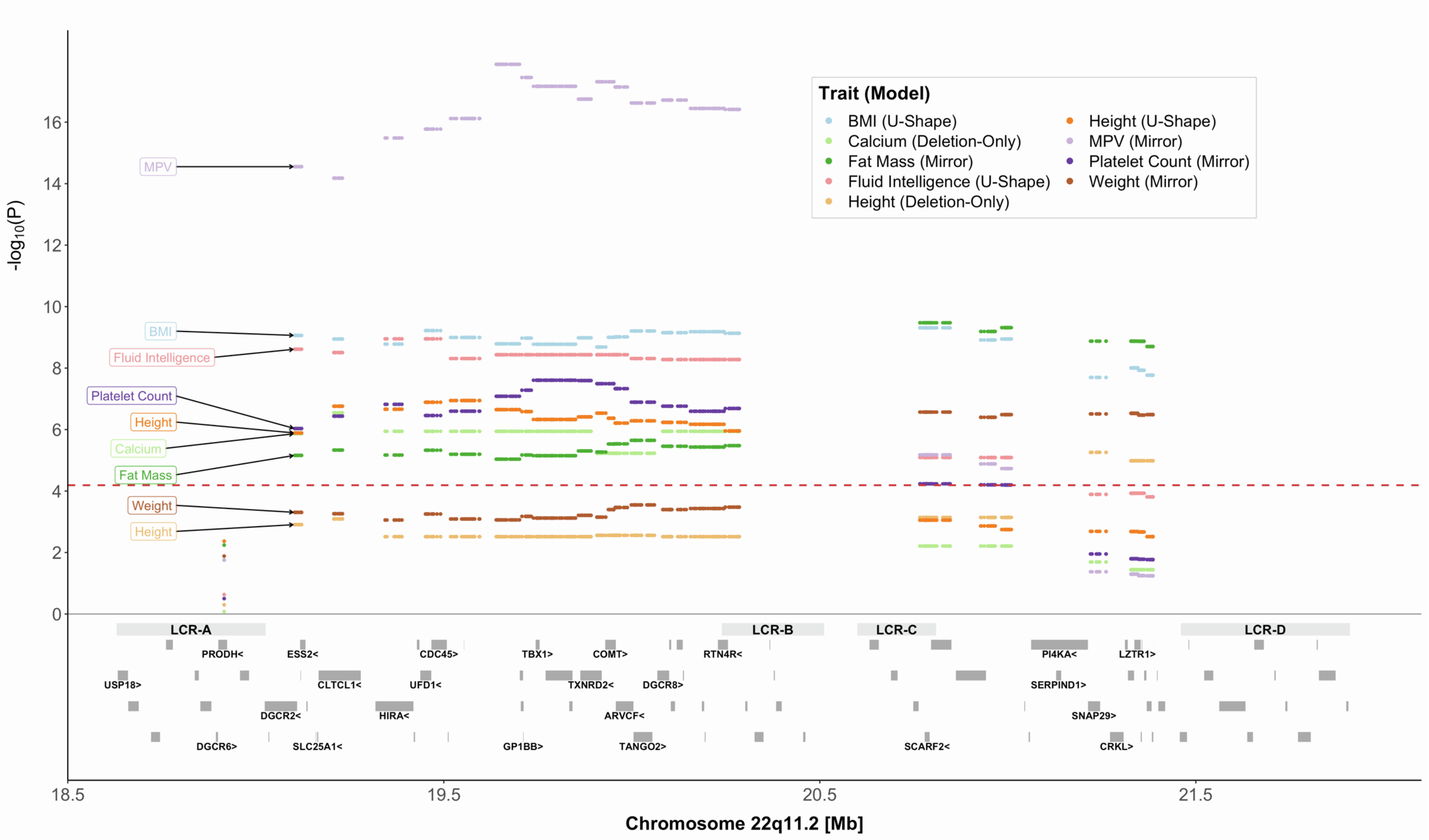

Trait (Model)
BMI (U-Shape)
Calcium (Deletion-Only)
Fat Mass (Mirror)
Fluid Intelligence (U-Shape)
Height (Deletion-Only)
Height (U-Shape)
MPV (Mirror)
Platelet Count (Mirror)
Weight (Mirror)
MPV
BMI
Fluid Intelligence
Platelet Count
Height
Calcium
Fat Mass
Weight
Height
-log10(P)
0
2
4
6
8
10
12
14
16
LCR-A
LCR-B
LCR-C
LCR-D
PRODH<
ESS2<
CDC45>
TBX1>
COMT>
RTN4R<
PI4KA<
LZTR1>
USP18>
CLTCL1<
UFD1<
TXNRD2<
DGCR8>
SERPIND1>
DGCR2<
HIRA<
ARVCF<
SNAP29>
DGCR6>
SLC25A1<
GP1BB>
TANGO2>
SCARF2<
CRKL>
18.5
19.5
20.5
21.5
Chromosome 22q11.2 [Mb]

Figure S2 | 22q11.2 CNVs and significant continuous traits. Top: The negative logarithm of the association p-value for the CNV-trait association for the model indicated in the legend is plotted against the 22q11.2 genomic region. Each point represents a CNV proxy probe. The red dashed line indicates significance threshold ($p < 6.5 \times 10^{-5}$). **Bottom:** Gray bars represent low copy-repeat region (LCR) A-D, as well as the 90 genes contained in the region. The 24 genes used for trait selection are labeled in black.

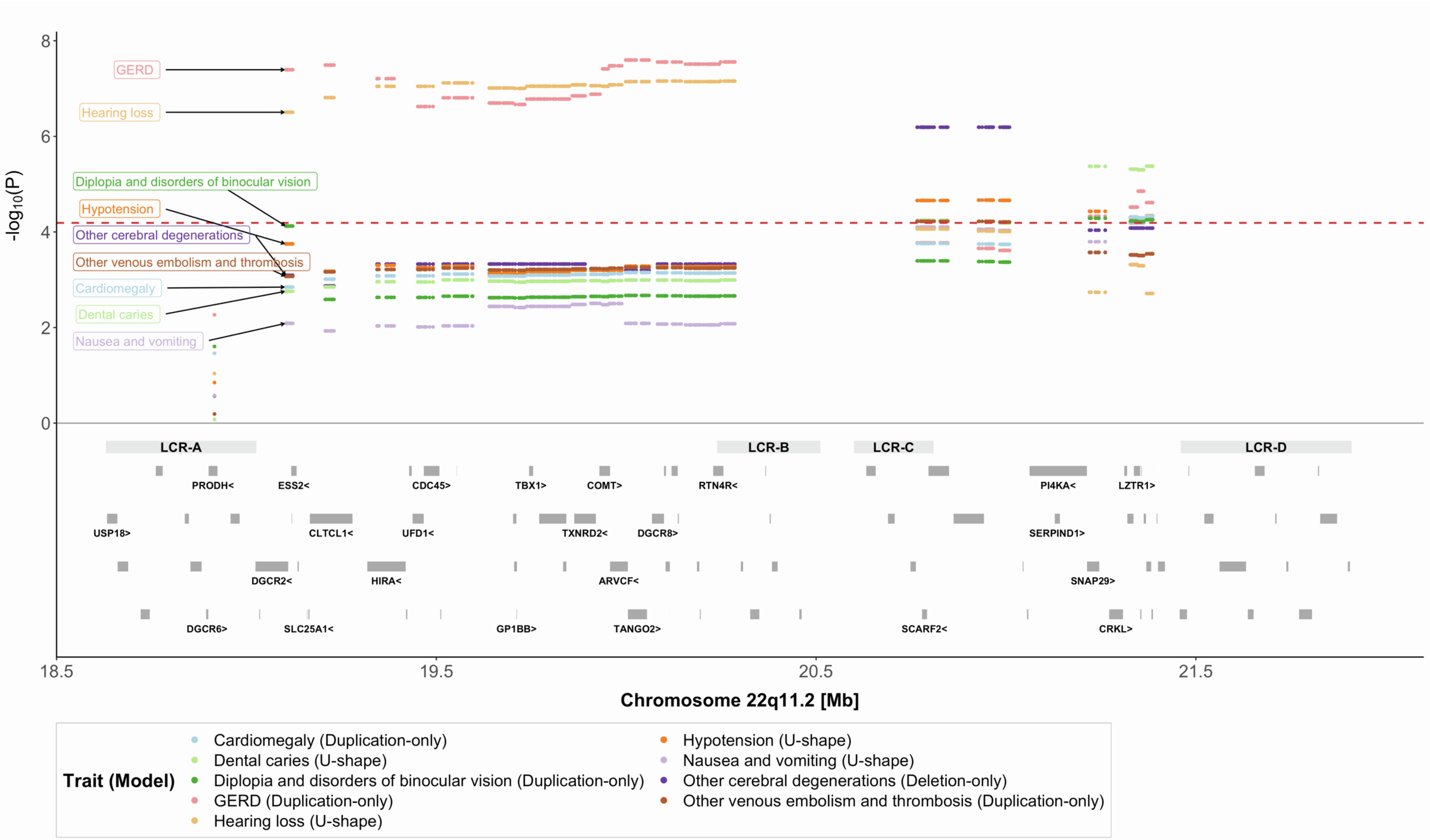

GERD
Hearing loss
Diplopia and disorders of binocular vision
Hypotension
Other cerebral degenerations
Other venous embolism and thrombosis
Cardiomegaly
Dental caries
Nausea and vomiting
-log10(P)
8
6
4
2
0
LCR-A
LCR-B
LCR-C
LCR-D
PRODH<
ESS2<
CDC45>
TBX1>
COMT>
RTN4R<
PI4KA<
LZTR1>
USP18>
CLTCL1<
UFD1<
TXNRD2<
DGCR8>
SERPIND1>
DGCR2<
HIRA<
ARVCF<
SNAP29>
DGCR6>
SLC25A1<
GP1BB>
TANGO2>
SCARF2<
CRKL>
18.5
19.5
20.5
21.5
Chromosome 22q11.2 [Mb]
Trait (Model)
Cardiomegaly (Duplication-only)
Dental caries (U-shape)
Diplopia and disorders of binocular vision (Duplication-only)
GERD (Duplication-only)
Hearing loss (U-shape)
Hypotension (U-shape)
Nausea and vomiting (U-shape)
Other cerebral degenerations (Deletion-only)
Other venous embolism and thrombosis (Duplication-only)

Figure S3 | 22q11.2 CNVs and significant binary traits. Top: The negative logarithm of the association p-value for the CNV-trait association for the model indicated in the legend is plotted against the 22q11.2 genomic region. Each point represents a CNV proxy probe. The red dashed line indicates significance threshold ($p < 6.5 \times 10^{-5}$). **Bottom:** Gray bars represent low copy-repeat region (LCR) A-D, as well as the 90 genes contained in the region. The 24 genes linked to traits according to HPO are labeled in black.

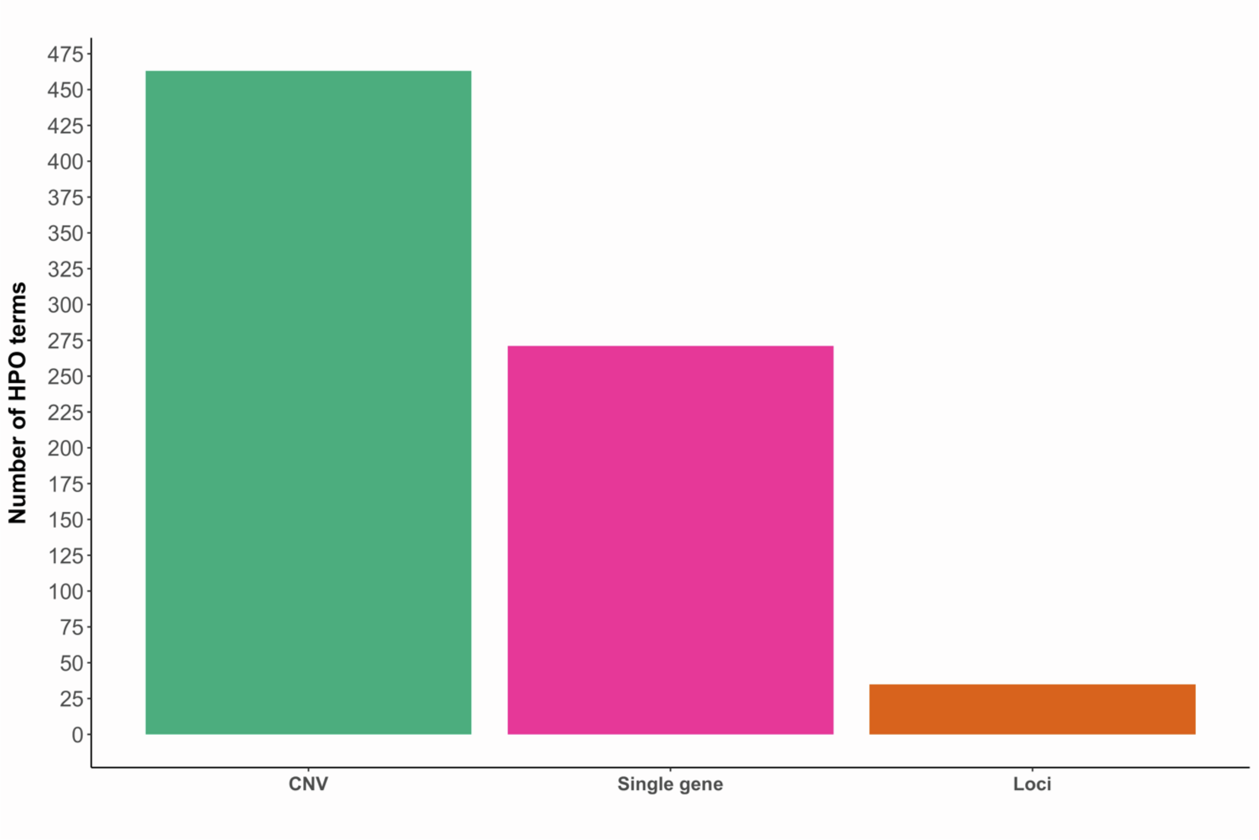

Figure S4 | HPO terms linked to 22q11.2 genes through different genetic variants. Barplot showing the number of HPO terms used in this study linked to conditions caused by 22q11.2 CNVs (green), genetic variants affecting a single gene in the region (pink) or linked to the 22q11.2 gene loci (orange).
